# Functional and taxonomic shifts in rhizosphere microbiomes of summer legume cover crops

**DOI:** 10.3389/fpls.2026.1790810

**Published:** 2026-05-01

**Authors:** Sri Kiran Reddy Alla, Vijay Joshi

**Affiliations:** 1 Department of Horticultural Sciences, Texas A&M University, College Station, TX, United States; 2 Texas A&M AgriLife Research and Extension, Uvalde, TX, United States

**Keywords:** amplicon sequencing, bacterial communities, cover crops, legumes, rhizobiome, soil microbial communities, sustainable agriculture

## Abstract

Legume cover crops are increasingly incorporated into sustainable production systems, yet their influence on rhizosphere microbial communities remains poorly characterized, particularly for underutilized warm-season species. In this study, we investigated rhizosphere bacterial communities associated with cowpea (*Vigna unguiculata*), tepary bean (*Phaseolus acutifolius*), and sunn hemp (*Crotalaria juncea*) in an organic production system using 16S rRNA amplicon sequencing and compared them with fallow soils. All legume species were associated with shifts in bacterial community composition relative to fallow soil, with increased relative abundance of copiotrophic taxa such as *Proteobacteria*, while fallow soils were dominated by oligotrophic groups within *Actinobacteriota*. Distinct species-specific recruitment patterns were observed: tepary bean rhizospheres showed higher relative abundance of *Rhizobiales*, associated with taxa involved in biological nitrogen fixation, whereas cowpea and sunn hemp supported bacterial groups linked to organic matter turnover and plant growth. Weighted UniFrac analyses indicated clear separation between cover-cropped and fallow soils, accounting for approximately 54% of community variation. Functional predictions using PICRUSt2 suggested representation of pathways related to central carbon metabolism, amino acid biosynthesis, and siderophore production under legume rhizospheres. Predicted pathways associated with nitrogen metabolism also tended to be more represented under cover crops, although no pathways remained significant after false discovery rate correction. Overall, these findings indicate that summer legume cover crops are associated with shifts in rhizosphere bacterial communities and predicted functional potential relative to fallow soils. These findings highlight the potential of species selection in shaping rhizosphere microbial communities in organic production systems and provide a foundation for future studies investigating microbial processes associated with cover cropping.

## 1 Introduction

The rhizosphere—the narrow soil region influenced by plant roots—is a dynamic interface central to plant health and ecosystem function. It hosts diverse microbial communities sustained by root exudates, which supply carbon and shape microbial assembly (Bakker et al., 2013; Yang et al., 2025). These microbes regulate nutrient acquisition, contribute to abiotic and biotic stress tolerance, and suppress soilborne pathogens (Agyekum et al., 2023), leading to the recognition of the rhizosphere microbiome as an extension of the plant’s ‘second genome’ (Li et al., 2021). Root exudates serve as biochemical cues that modulate microbial recruitment, and stress conditions further alter exudate composition, selectively attracting beneficial taxa (Fan et al., 2025; Wankhade et al., 2025).

Legumes (Fabaceae) play a critical role in agroecosystems by supplying protein-rich food and improving soil fertility through symbiotic nitrogen fixation (Schaedel et al., 2021). This process relies on coordinated signaling between plant-derived flavonoids and rhizobial Nod factors, which initiate nodule formation (Schaedel et al., 2021; Liu and Murray, 2016). However, symbiotic efficiency varies widely depending on rhizobial competitiveness, host genotype, and environmental conditions (Han et al., 2020; Xiao et al., 2017). Beyond rhizobia, a diverse assemblage of non-symbiotic microbes inhabits the rhizosphere and nodules, contributing to nutrient cycling, stress resilience, and plant development (Qiao et al., 2024; Nysanth et al., 2025). Recent studies suggest that these microbiomes form complex ecological networks shaped by host identity, root architecture, and exudate composition, often leading to species-specific recruitment patterns (Park et al., 2023; Pérez-Jaramillo et al., 2017). For example, cowpea roots harbor diverse non-rhizobial endophytes that are influenced by soil type (Leite et al., 2017), whereas sunn hemp can alter microbial community composition and function in ways distinct from those in non-legume systems (Eo et al., 2015; Leite et al., 2021). In contrast, tepary bean (*Phaseolus acutifolius*), a drought-tolerant legume of increasing relevance for climate-resilient agriculture, remains comparatively understudied with respect to rhizosphere microbial dynamics (Traub et al., 2017).

Cover cropping is widely adopted to improve soil health during fallow periods by enhancing nutrient retention, increasing soil organic matter, and promoting soil structure and biodiversity (Blanco-Canqui et al., 2015; Shrestha et al., 2002; Bergtold et al., 2017). Legume cover crops are particularly valuable because biological nitrogen fixation and their rhizodeposition can stimulate microbial activity and increase soil fertility (Zhang et al., 2025; Wooliver et al., 2025). Root exudates play a central role in these interactions, providing carbon substrates and signaling compounds that shape rhizosphere microbial communities and regulate functions such as nutrient mineralization, organic matter turnover, and disease suppression (Chaparro et al., 2013; Seitz et al., 2024). Despite these benefits, the extent to which different legume species recruit distinct microbial assemblages remains poorly understood, particularly for underutilized warm-season legumes relevant to organic production systems.

Advances in high-throughput sequencing, particularly 16S rRNA amplicon sequencing, have enabled characterization of rhizosphere microbial communities and their potential functional roles in soil ecosystems (Zhang et al., 2021; Licata et al., 2025). These approaches allow researchers to investigate microbial diversity and infer potential metabolic functions associated with plant-soil interactions (Regueira-Iglesias et al., 2023; Butler et al., 2025).

Although legumes share key functional traits, their rhizosphere microbiomes can differ substantially among species (Cazzaniga et al., 2023). Extensive research has studied microbial dynamics in common legumes such as soybean, common bean, and cowpea (de Albuquerque et al., 2022; Jaiswal et al., 2021; Han et al., 2020; Pérez-Jaramillo et al., 2017), but comparatively little is known about microbiome assembly in climate-resilient species like tepary bean (Porch et al., 2024; Singh et al., 2024). In this study, we evaluated cowpea (*Vigna unguiculata*), sunn hemp (*Crotalaria juncea*), and tepary bean (*Phaseolus acutifolius*) as warm season cover crops in an organic production system. These species differ in biomass production, nitrogen contributions, and ecological functions, with cowpea and sunn hemp widely recognized for their soil-building properties and associated microbial symbionts (Clark, 2012; Zahran, 2001; Peoples et al., 2009), but mechanisms behind species-specific recruitment and functional changes in warm-season legumes are less understood than in major legumes. This gap hinders the development of climate-resilient cover crops for warm environments (Cazzaniga et al., 2023; Singh et al., 2024). Rhizosphere−focused 16S rRNA sequencing offers the taxonomic detail needed to detect species−level assembly patterns that respond strongly to root-derived carbon inputs. Recent research indicates that legume rhizodeposition improves nitrogen cycling and that cover crop root exudates can shape microbiome functional paths (Yang et al., 2024; Qiao, 2024; Seitz et al., 2024; Tao et al., 2024). However, direct comparisons of summer legumes remain limited, underscoring the need for an integrated taxonomic and functional approach to evaluate tepary bean, cowpea, and sunn hemp. We hypothesized that each legume species would assemble a distinct rhizosphere microbial community, distinguished not only by taxonomic differences but also by notable shifts in (i) alpha-diversity (richness and evenness), (ii) beta-diversity (abundance-weighted and phylogeny-based community structure), and (iii) predicted functional capabilities, especially pathways related to carbon metabolism and nitrogen transformation. Clarifying these expectations helps in better understanding species-specific microbial recruitment and the functional trends observed in subsequent analyses.

## 2 Materials and methods

### 2.1 Site, experimental design, and plant materials

**Figure 1:**
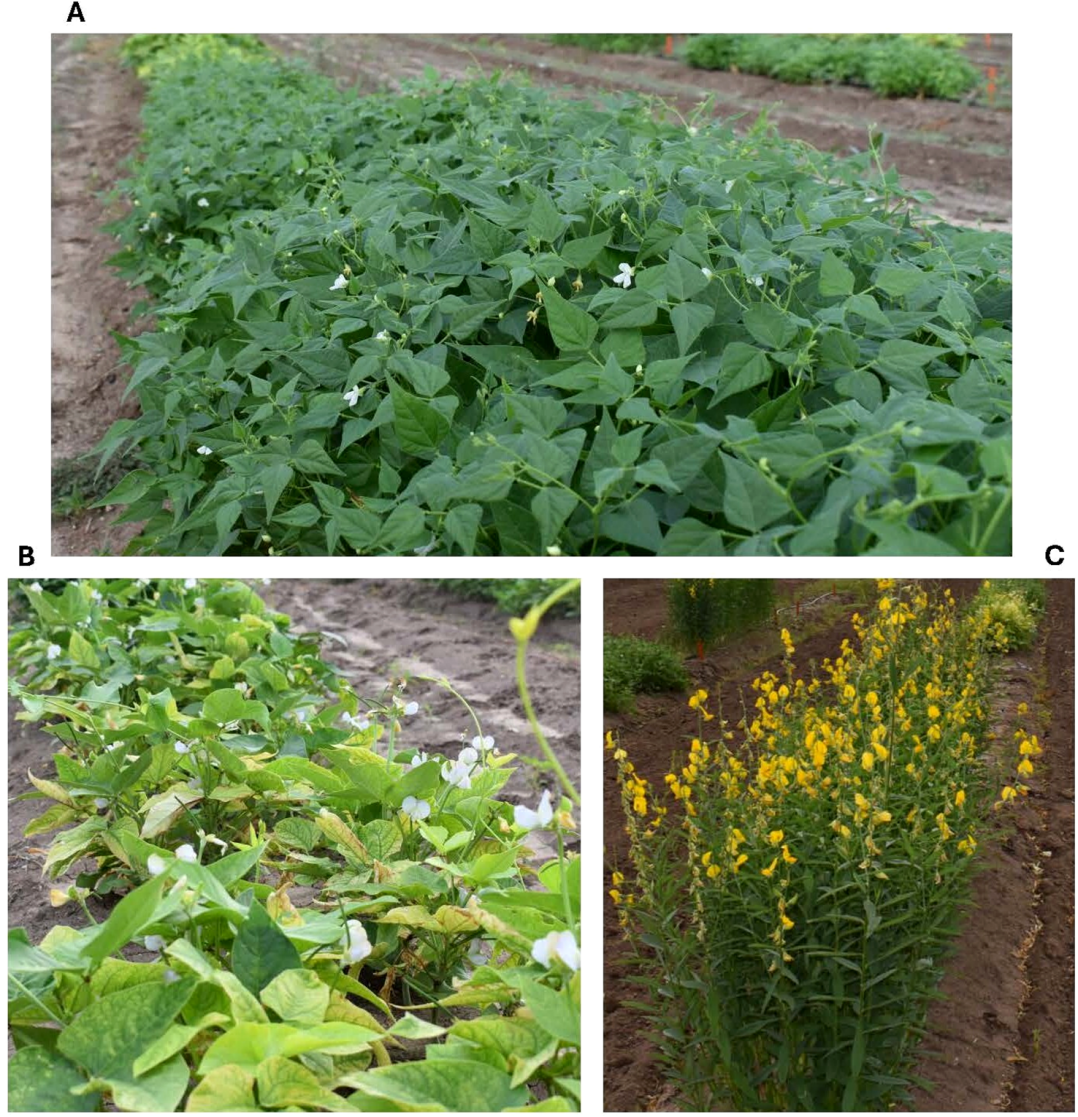
Legume cover crop species. **(A)** Tepary cover, **(B)** Cowpea cover, **(C)** Sunn hemp cover.

The field study was conducted in a certified organic field at the Texas A&M AgriLife Research and Extension Center (Uvalde, Texas, USA). The study was arranged in a randomized complete block design (RCBD) with three replications/blocks. Each block was 250 ft × 2 ft, and in each block, three cover crop treatments (**Figure 1**): tepary bean (*Phaseolus acutifolius;* Cultivar: Sacaton brown), cowpea (*Vigna unguiculata;* Cultivar: Californian Blackeye46), and sunn hemp (*Crotalaria juncea;* Cultivar: Crescent sunn)—and an untreated fallow control without vegetation were arranged. Individual plots within each block measured 15 ft × 2 ft, with a 3 ft buffer between plots. Seeds were broadcast uniformly within each plot and lightly incorporated into the soil to promote adequate soil contact and germination. The seeding rate was 35-40 g per plot for cowpea and tepary bean, and 20-25 g per plot for sunn hemp. No surface sterilization of seeds was performed, as this was a cover crop study conducted under sustainable, low-input agricultural systems; all plots were managed according to standard organic practices, such as avoiding synthetic fertilizers and herbicides, and organic management strategies, such as using Organic Materials Review Institute (OMRI)-certified biopesticides.

### 2.2 Baseline soil analysis and biomass of cover crop species

Soil analysis was conducted by the Phytonutrient Laboratory, Texas A&M AgriLife Research, Uvalde, TX, which reported soil nitrate concentrations as NO_3_^-^–N (% of dry soil mass). Soil samples were collected to determine baseline nutrient status before cover crop sowing and to evaluate soil nitrate availability following cover crop decomposition. For baseline soil analysis, the field consisted of three rows, each representing an experimental replication. Within each row, 5 to 6 soil cores were randomly collected and pooled into a single composite sample, resulting in 3 composite samples across the three replications. These samples were analyzed to determine initial soil nitrate (NO_3_^-^–N%) and other soil chemical properties. After cover crop termination and residue decomposition, soil samples were collected again to assess soil nitrate levels. At this stage, each row contained four plots (cowpea, tepary, sunn hemp, and fallow). From each plot, 3 to 4 soil cores were collected and pooled into a single composite sample, yielding one composite soil sample per plot for laboratory analysis.

Additionally, the aboveground biomass of each cover crop species was measured. 3 individual plants from each plot were selected randomly to measure above-ground fresh biomass. The same samples were oven-dried at 70 °C for 4-5 days to record dry biomass.

### 2.3 Rhizosphere sampling and handling

Sampling was conducted 60 days after sowing. Representative plants were gently uprooted to preserve the soil adhering to the roots. Loosely attached soil was removed by gentle shaking. Belowground roots were excised into sterile bags on ice. Rhizosphere material was collected from 3-4 representative plants within each plot and pooled into a single composite sample. For fallow plots, bulk soil cores (3–5 per subplot) were composited. Samples were stored at −80 °C. Sterile technique was maintained throughout field and lab handling.

### 2.4 Rhizosphere fraction recovery and DNA extraction

We used 20X PBS (phosphate-buffered saline; 1X 0.01M Na Phosphate, 0.15M NaCl) from Thermo Scientific and diluted it to 1X PBS (To make 1 L of 1X PBS, 50 mL of 20X PBS was mixed with 950 mL of distilled water). Frozen samples were thawed on ice and transferred to sterile 50 mL tubes containing 1× PBS. Tubes were vortexed briefly (30 s, low speed) and sonicated (30 s) to detach rhizosphere material from roots. Following sonication, roots were removed using sterile forceps. The soil suspension was centrifuged at 3500 × g for 5 min at 4 °C, and the supernatant was discarded. The resulting pellet constituted the rhizosphere fraction for DNA extraction.

Total DNA was extracted using the Qiagen DNeasy PowerSoil Pro Kit according to the manufacturer’s instructions with minor optimizations. In addition to the manufacturer’s protocol, we included an additional C5 wash and an empty, dry spin before eluting DNA. DNA concentration and purity were assessed with a DeNovix DS-11 spectrophotometer, with acceptance criteria of ~1.7–1.9 for the A260/A280 ratio. Extracted DNA was immediately stored at −20 °C before library preparation.

### 2.5 16S rRNA gene sequencing and bioinformatic analysis

Bacterial community profiling was performed using the Quick-16S™ NGS Library Prep Kit (Zymo Research, Irvine, CA). Library preparation targeted the V3-V4 region of the 16S rRNA gene using custom primers: Forward primer 341F (5′-CCTAYGGGDBGCWGCAG-3′) and Reverse primer 806R (5′-GAMTACNVGGGTHTCTAATCC-3′). To minimize amplification of plant-derived sequences, chloroplast (pPNA) and mitochondrial (mPNA) peptide nucleic acid (PNA) blockers were included during PCR to suppress chloroplast and mitochondrial 16S rRNA amplification.

Indexed libraries were prepared and sequenced by Zymo Research, Irvine, CA, on an Illumina^®^ NextSeq™ platform using a P1 reagent kit (600 cycles, paired-end), with a 30% PhiX spike-in to increase sequence diversity. Raw sequencing reads were processed using the DADA2 pipeline (Callahan et al., 2016). Briefly, reads were quality-filtered and trimmed, error models were constructed, amplicon sequence variants (ASVs) were inferred, and chimeric sequences were identified and removed. The resulting high-resolution ASV table was used for all downstream analyses.

Taxonomic assignment (Oren and Garrity (2021) was performed using UCLUST within QIIME v1.9.1 (Caporaso et al., 2010), using the Zymo Research reference database, which was curated based on the SILVA and Greengenes databases. Community composition analyses, including relative abundance bar plots and heatmaps at multiple taxonomic levels (phylum, class, order, family, genus, and species), were generated using QIIME and Zymo Research internal scripts. Sequencing depth, total reads after chimera filtering, and final ASV counts are reported in the Results section.

### 2.6 Alpha- and beta-diversity analyses

Alpha- and beta-diversity analyses were conducted within QIIME v1.9.1. Principal coordinate analysis (PCoA) plots were generated to visualize community dissimilarities. To assess sequencing depth sufficiency and within-sample diversity, alpha rarefaction curves were generated from rarefied ASV tables. Alpha diversity metrics included observed richness, the Chao1 index, and the Shannon diversity index. Groupwise differences were evaluated using the Kruskal–Wallis test followed by Benjamini–Hochberg–adjusted Dunn’s *post hoc* tests; effect sizes were estimated using Cliff’s δ and Hedges’ g.

Between-sample (beta) diversity was assessed using weighted UniFrac distance matrices generated by the Zymo Research bioinformatics pipeline (Caporaso et al., 2010). Phylogeny-based distance calculations were performed by Zymo Research using representative ASV sequences. The resulting distance matrices were provided and imported into R for statistical analysis and visualization. Principal Coordinate Analysis (PCoA) was used to visualize community structure. Ellipses represent 95% confidence intervals around group centroids, calculated using stat_ellipse (ggplot2).

Overall differences among treatments were tested using PERMANOVA (adonis2, 999 permutations) and implemented in the vegan package in R (version 2.7-2; Oksanen et al., 2013). Homogeneity of multivariate dispersion was evaluated using the betadisper function in the vegan package to confirm that differences in within-group dispersion did not drive our PERMANOVA results. Comparisons included all treatments and pooled cover crop treatments versus the fallow control. Pairwise PERMANOVA analyses were conducted using (pairwise.Adonis) to evaluate contrasts between individual cover crops and the fallow control. Complementary dissimilarity metrics, including Bray-Curtis and unweighted UniFrac distances, were used for analysis.

### 2.7 Differential taxonomy and biomarker analyses

Differentially abundant taxa were identified at multiple taxonomic ranks using linear discriminant analysis effect size (LEfSe) (Segata et al., 2011), with thresholds of LDA > 3 and p < 0.05. LEfSe was used to detect bacterial biomarkers that best characterize each cover crop rhizosphere relative to fallow soil and among cover crops. In addition, genus-level differential abundance patterns were summarized to highlight taxa that were enriched or depleted under each treatment. To clarify our analytical workflow, we employed LEfSe and GLM-based differential abundance analyses to complement each other. LEfSe (LDA > 3, p < 0.05) served as an initial biomarker−screening tool to identify taxa with significant discriminatory power among treatments. We recognize that LEfSe lacks compositional awareness; thus, biomarkers identified by LEfSe were cautiously interpreted and cross-validated with GLM trends. This limitation aligns with broader findings of variability among differential−abundance tools (e.g., Nearing et al., 2022), and future studies could explicitly incorporate compositional methods such as ANCOM−BC or ALDEx2 to confirm the results further.

### 2.8 Functional prediction of bacterial communities

Functional profiles were predicted using PICRUSt2 (Douglas et al., 2019) based on the observed ASV profiles. MetaCyc pathway abundances were inferred from gene copy number predictions and summarized as mean values per treatment. To visualize pathway-level differences across treatments, row-scaled (z-score) heatmaps were generated for top predicted pathways and MetaCyc superclass summaries. Emphasis was placed on nitrogen transformation pathways (e.g., denitrification and assimilatory nitrate reduction), central carbon metabolism (e.g., the TCA cycle and carbohydrate metabolism), amino acid and nucleotide biosynthesis, fatty acid, and lipid biosynthesis, siderophore biosynthesis, and aromatic compound degradation. Nonparametric tests evaluated group differences; where applicable, false discovery rate (FDR) control was applied.

PICRUSt2 QC and database details: ASV representative sequences were placed with EPA-NG/hmmalign under the default pipeline, and NSTI was computed per ASV and sample. We report sample-weighted NSTI (mean ± SD) and the fraction of ASVs with NSTI ≤0.20. We verified MetaCyc minpath pathway coverage and per-sample placement success. Predictions were generated using PICRUSt2 and the PICRUSt2-SC reference database (v2.6.0+), which expands the representation of bacterial and archaeal genomes for environmental microbiomes. Interpretations acknowledge the limits of inference-based predictions in soils.

### 2.9 Data availability

All sequencing data have been deposited in the National Center for Biotechnology Information (NCBI) under BioProject accession PRJNA1337659.

## 3 Results

### 3.1 Soil analysis and biomass of cover crops

Soil chemical analysis was conducted by the Phytonutrient Laboratory, Texas A&M AgriLife Research, Uvalde, TX, which reported soil nitrate concentrations as NO_3_^-^–N (% of dry soil mass). Initial measurements ranged from 0.0042 to 0.0088% NO_3_^-^–N across samples. A follow-up analysis conducted after cover crop decomposition showed NO_3_^-^–N concentrations ranging from 0.0010 to 0.0061%, indicating variation in soil nitrate availability following residue breakdown.

Substantial variation was observed above-ground among the summer legume cover crops. Tepary produced the highest biomass, with fresh biomass ranging from 67 to 217.5 g/plant and dry biomass ranging from 23 to 57.5 g/plant, with mean values of 121.9 ± 37.8 g fresh weight and 35.7 ± 9.7 g dry weight per plant. Sunn hemp produced biomass ranged from 55 to 121.5 g/plant for fresh biomass and 21.5 to 51 g/plant for dry biomass, averaging 78.5 ± 24.2 g fresh weight and 33.1 ± 9.8 g dry weight per plant. Cowpea produced comparatively lower biomass, with fresh biomass ranging from 39.5 to 93.5 g/plant and dry biomass ranging from 15.5 to 31.5 g/plant, with mean values of 57.8 ± 16.8 g fresh weight and 21.4 ± 5.2 g dry weight per plant. Details of soil and biomass values were provided in **Supplementary Table 1**.

### 3.2 Sequencing output and ASV recovery

Amplicon sequencing of nine rhizosphere samples representing cowpea, tepary bean, and sunn hemp, along with three bulk soil controls, generated a total of 2,309,296 raw reads. After quality filtering and trimming, 993,016 reads were retained for error modelling and denoising using the DADA2 pipeline. Removal of 38,531 chimeric sequences resulted in 954,484 high-quality reads for downstream analysis. Across all samples, a total of 17,924 unique amplicon sequence variants (ASVs) were identified.

**Figure 2:**
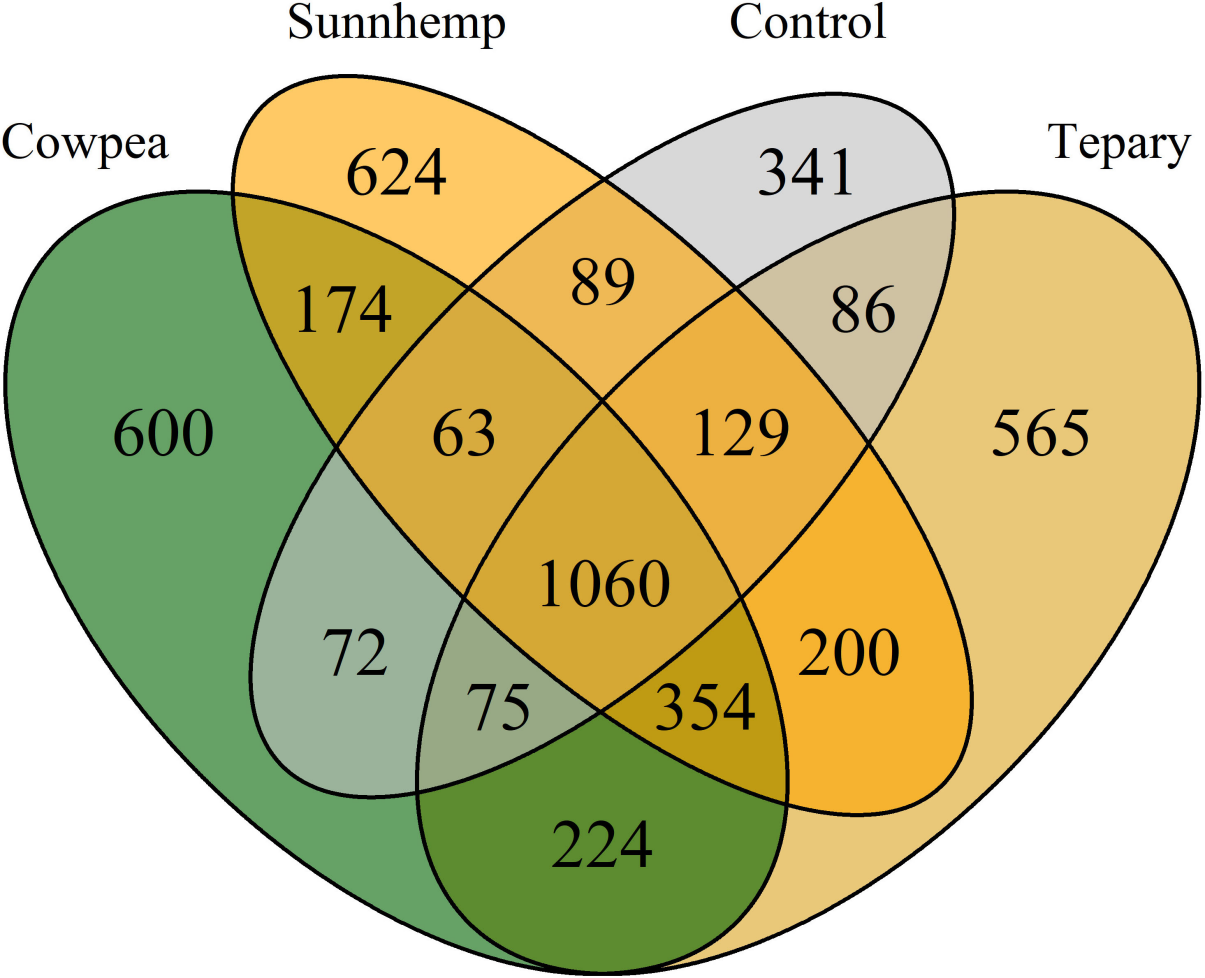
Venn diagram of amplicon sequence variants (ASVs) of bacterial communities across cover crop rhizospheres and fallow soil. A total of 1060 shared ASVs were observed across all samples, and 600, 624, 565, and 341 ASVs were unique to cowpea, sunn hemp, tepary, and fallow.

Sequencing depth was comparable across treatments, as indicated by the number of retained reads per sample. Microbial diversity, measured as ASV richness, also showed similar ranges across crop treatments. Sunn hemp samples exhibited the highest ASV richness (1,413–1,911 ASVs), followed by tepary bean (1,259–1,905 ASVs), cowpea (1,065–1,905 ASVs), and fallow soil (983–1,351 ASVs). Community overlap analysis indicated substantial taxon sharing among treatments. A Venn diagram revealed 1,060 ASVs shared across all treatments, while 600, 565, 624, and 341 ASVs were uniquely detected with cowpea, tepary bean, sunn hemp, and fallow soils, respectively (**Figure 2**; **Supplementary Table 2**). These results suggest the presence of a broad bacterial taxa across treatments, along with species-specific microbial assemblages that may reflect differences in root traits and rhizosphere exudation patterns among legumes.

### 3.3 Alpha diversity

**Figure 3:**
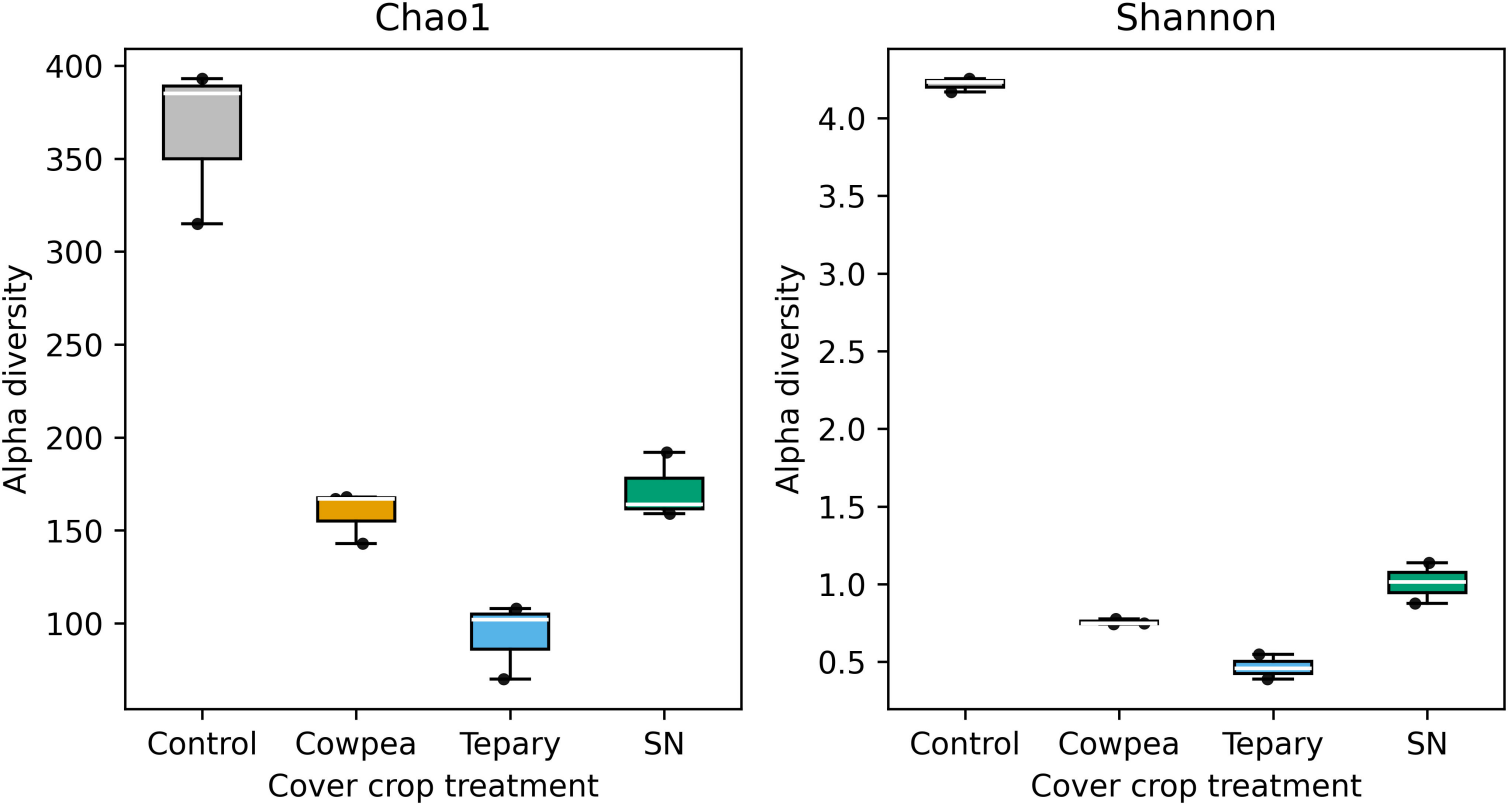
Alpha diversity (Chao1 and Shannon) across cover−crop treatments. Boxplots (coloured by treatment) show the distributions of Chao1 richness and Shannon diversity for Control (no cover), Cowpea, Tepary, and SN (Sunn hemp), with all individual samples overlaid as jittered points. Group differences were assessed using a Kruskal–Walli’s test (Chao1: p = 0.0249; Shannon: p = 0.0156). Pairwise Mann–Whitney U contrasts versus Control were corrected with the Benjamini–Hochberg FDR; for both metrics, Cowpea, Tepary, and SN showed q = 0.10. Corresponding non−parametric Cliff’s δ effect sizes for all contrasts were –1.00, indicating higher alpha−diversity values in the Control relative to the cover−crop treatments.

Rarefaction curves plateaued at sequencing depths ≥12,000 reads for all treatments, indicating adequate sampling depth. Alpha diversity did not differ significantly among treatments (Kruskal–Wallis: H = 3.31, df = 3, p = 0.347). Dunn *post hoc* tests with Benjamini–Hochberg adjustment yielded q ≥ 0.42 for all pairwise contrasts. Although rhizosphere communities showed slightly higher richness and evenness than fallow, these differences were not statistically significant (**Figure 3**; **Supplementary Figure 1**; **Supplementary Table 3**). Hence, we interpret alpha diversity patterns as exploratory trends and may need further validations to confirm the effect.

### 3.4 Beta diversity

**Figure 4:**
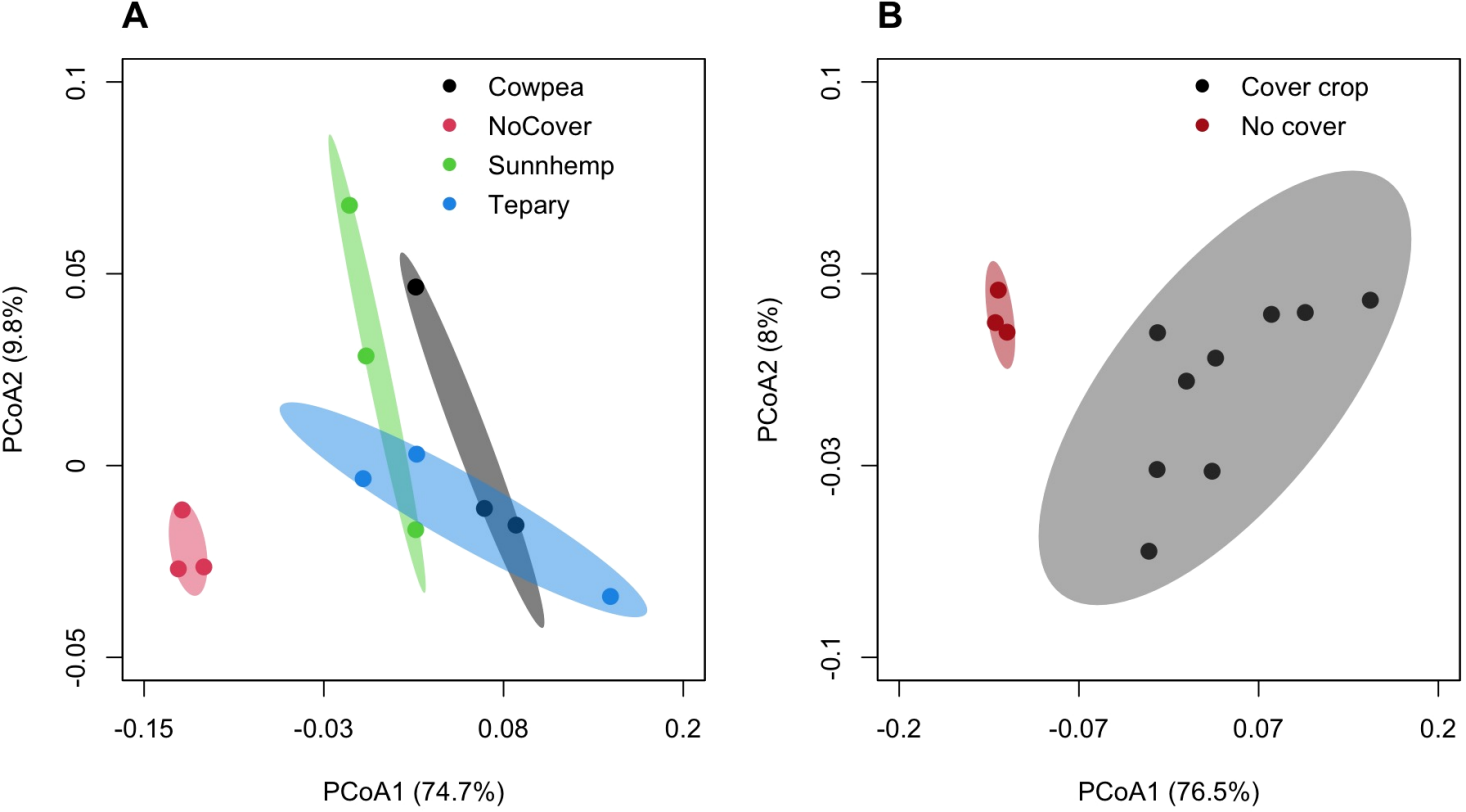
Principal coordinate analysis (PCoA) of bacterial communities based on weighted UniFrac distances from the rarefied ASV table; ellipses = 95% confidence intervals; n = 3 per treatment. **(A)** All treatments: PC1 = 74.7%, PC2 = 9.8%; PERMANOVA (999 permutations): F = 5.70, R² = 0.68, p = 0.003. **(B)** Cover crops vs. fallow: PERMANOVA (999 permutations): F = 12.08, R² = 0.55, p = 0.006.

Community structure differed between legume rhizospheres and fallow soil. Weighted UniFrac principal coordinate analysis (PCoA; **Figure 4**) showed separation among treatments. PERMANOVA testing of all treatments together indicated a significant overall community shift (999 permutations: F = 5.70, R² = 0.68, p = 0.003). A separate comparison between pooled legume rhizosphere samples and fallow soil also indicated significant divergence (F = 5.70, R² = 0.55, p = 0.006). To verify our PERMANOVA results, homogeneity of multivariate dispersion was evaluated using betadisper in the vegan package in R (version 2.7-2). This analysis indicated no significant differences in dispersion among treatments (permutation test: F = 0.66, p = 0.62; **Supplementary Table 4**), supporting the interpretation that PERMANOVA results primarily reflect differences in community composition rather than variation in within-group dispersion.

Bray–Curtis dissimilarities similarly supported compositional differences among treatments (F = 4.28, R² = 0.30, p = 0.006; **Supplementary Figure 2**). Unweighted UniFrac analyses showed separation with smaller effect sizes (**Supplementary Figure 3**), suggesting that treatment effects were driven primarily by shifts in the relative abundance of phylogenetically related taxa rather than widespread presence–absence turnover.

Pairwise PERMANOVA comparisons between individual legume treatments and fallow soil indicated moderate effect sizes (R² ≈ 0.71–0.84) but were not statistically significant (p ≈ 0.10), likely reflecting the limited number of biological replicates. Therefore, these results are presented as exploratory patterns rather than definitive treatment effects. In contrast, comparisons among the three legume rhizospheres showed smaller and non-significant differences (R² ≈ 0.09–0.39; p = 0.20–0.80). Together, these analyses suggest that summer legume cover crops influence rhizosphere microbial community structure compared with fallow soil. However, additional replication would be needed to confirm species-specific differences among legume treatments.

### 3.5 Community composition and species-specific recruitment

**Figure 5:**
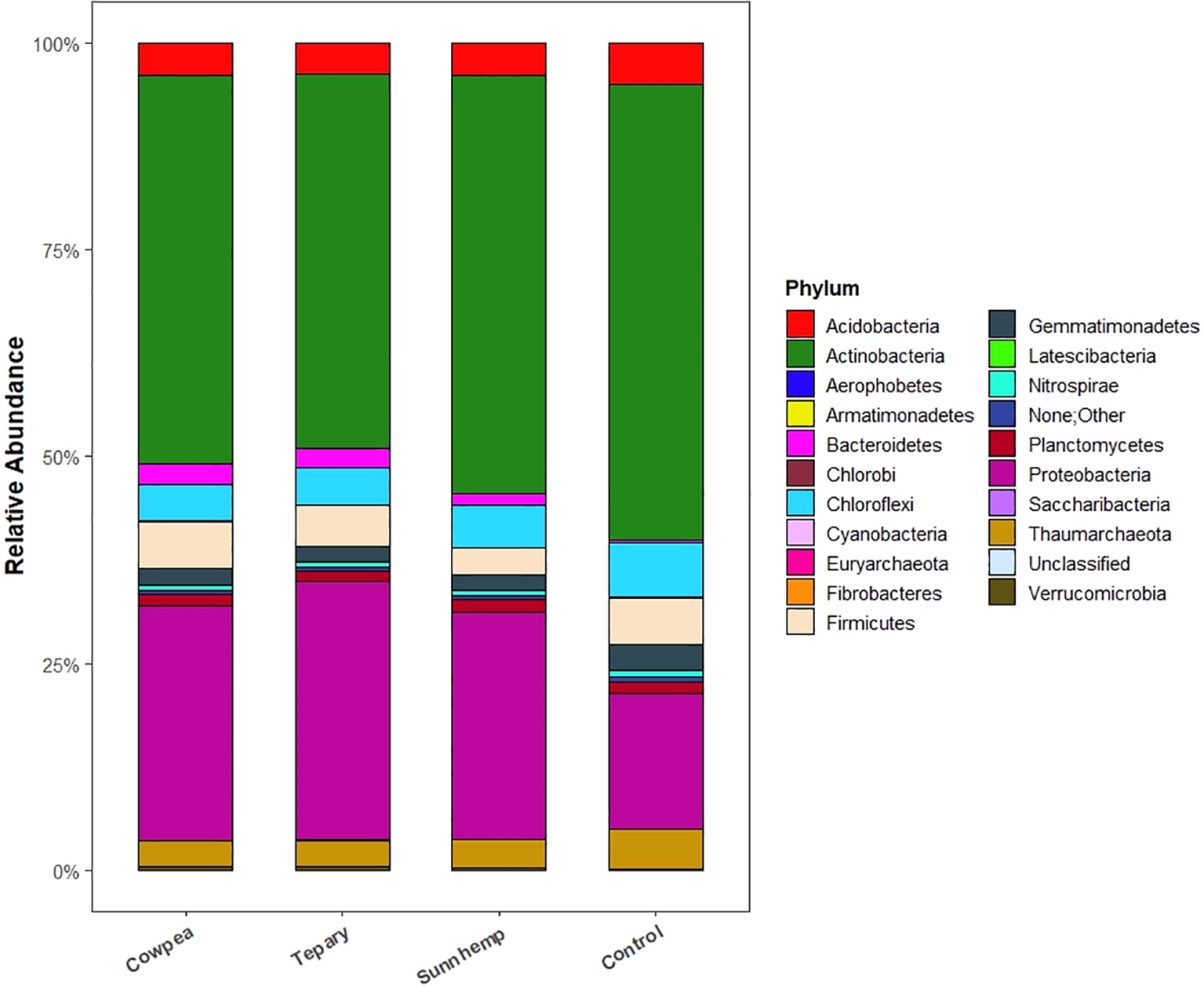
Phylum-level relative abundance of bacterial communities (top 25 phyla shown; remaining aggregated as “Other”). Values are relative abundances from the rarefied ASV table; n = 3 per treatment.

**Figure 6:**
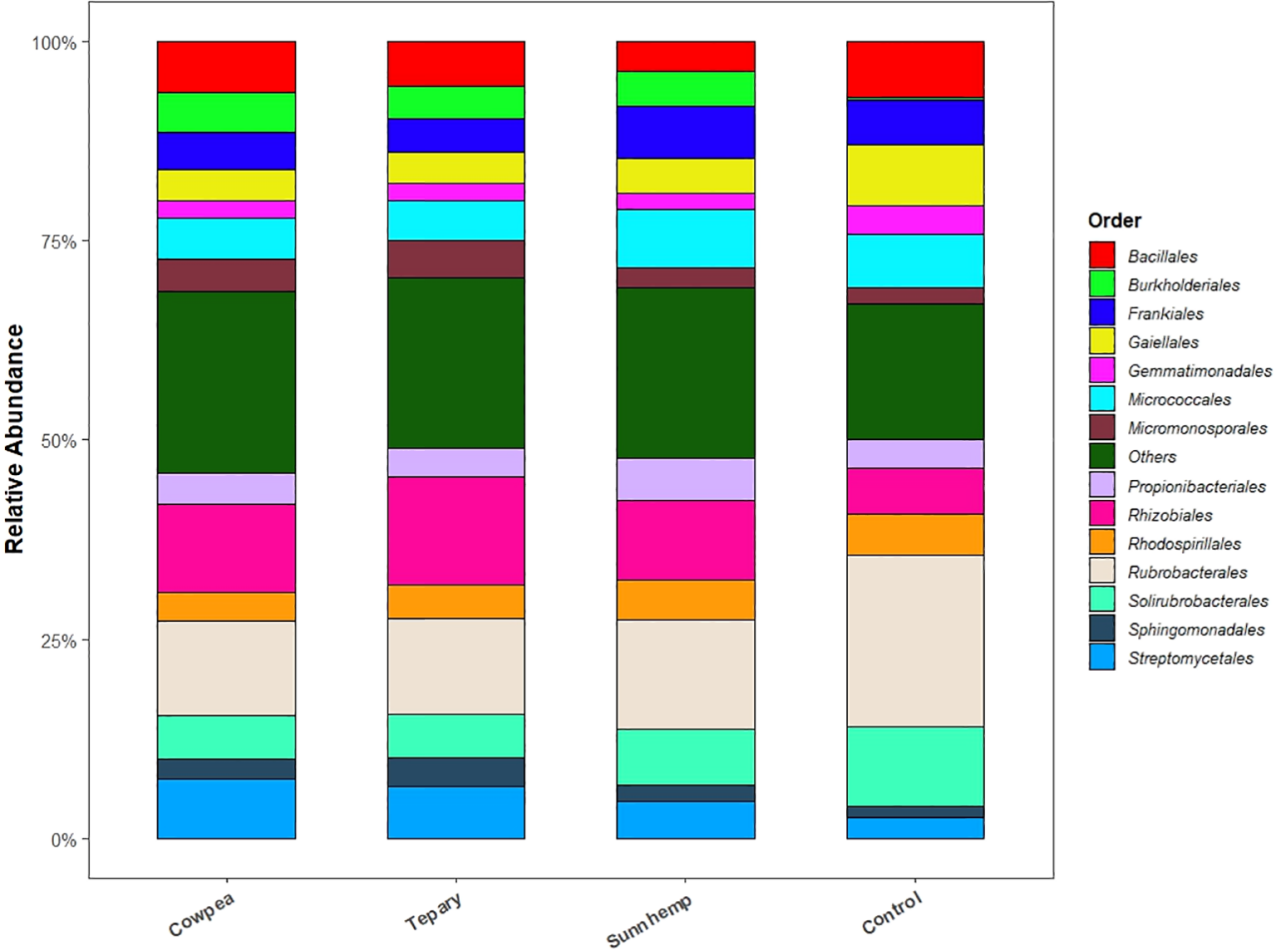
Order-level relative abundance of bacterial communities (top 25 orders shown; remaining aggregated as “Other”). Values are relative abundances from the rarefied ASV table; n = 3 per treatment.

**Figure 7:**
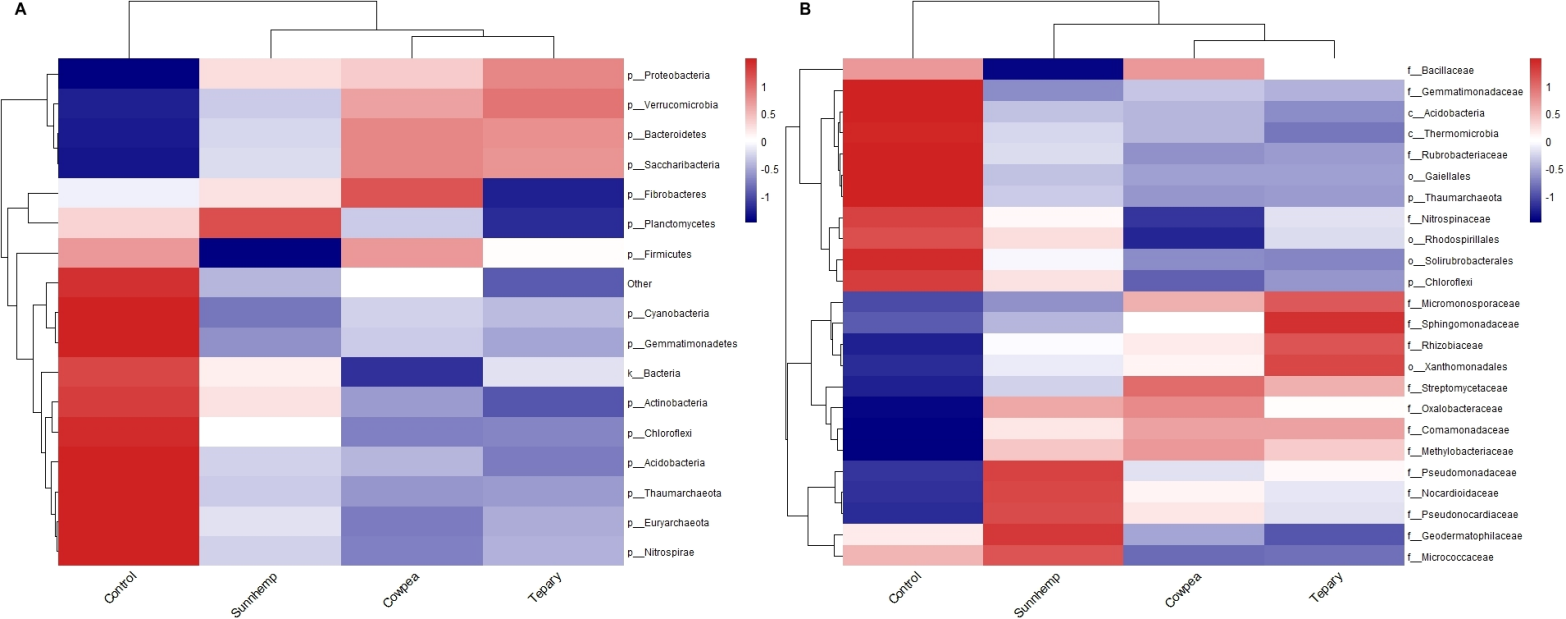
Heatmaps showing differential enrichment across treatments. **(A)** Phylum-level heatmap; **(B)** Family-level heatmap. Data are centered and scaled per taxon (row-scaled z-scores). Only the top 50 taxa by mean relative abundance are shown. n = 3 per treatment.

At the phylum level, a canonical rhizosphere shift from oligotrophy to copiotrophy was evident. Actinobacteriota dominated fallow soils (mean 55.38%) with classes such as *Rubrobacteria* (17.53%) and *Thermoleophilia* (14.44%), consistent with stress-tolerant, slow-growing taxa. In contrast, all legume rhizospheres were enriched in *Proteobacteria*—31.36% in tepary, 28.46% in cowpea, and 27.56% in sunn hemp (**Figures 5**, **6**)—reflecting recruitment of copiotrophic taxa favored by root-derived carbon inputs. At higher taxonomic resolution, species-specific assembly patterns emerged. Tepary bean strongly enriched *Rhizobiales* (11.83%), in line with legume-associated nitrogen-fixing partners and allied taxa. Cowpea favored *Streptomycetales* (6.5%) and *Burkholderiales* (4.33%), groups frequently linked to biocontrol, bioactive metabolite production, and decomposition of complex residues. Sunn hemp supported diverse *Actinobacteriota* associated with carbon turnover and stress adaptation. Conversely, fallow soil remained enriched in *Rubrobacterales* and other oligotrophic lineages (**Figures 6**, **7**).

Family-level profiles reinforced these patterns: tepary was enriched in *Rhizobiaceae*, *Sphingomonadaceae*, and *Micromonosporaceae*; cowpea in *Streptomycetaceae* and *Comamonadaceae*; sunn hemp in *Micrococcaceae* and *Pseudomonadaceae*; and fallow in *Rubrobacteriaceae* (**Figure 7**). Species-level signals cohered with these taxa. *Rubrobacter* sp. *10729* reached relative abundances of ~0.0085, consistent with adaptation to desiccation/stress; *Microlunatus* (to 0.0081), *Nocardioides* (to 0.0316; prominent in cowpea and tepary), *Kribbella* (~0.003–0.0037), and *Aeromicrobium* (~0.001–0.0026) implicated active carbon and phosphorus cycling. *Massilia* (0.008–0.019 in cowpea and tepary) suggested early root colonization capacity and stress tolerance. Low abundance nitrifiers (*Nitrosospira*, *Nitrososphaera*) indicate background nitrification potential. *Streptomyces* spp., including *S. sodiiphilus*, were present in tepary and sunn hemp, consistent with potential biocontrol and organic matter degradation roles (**Supplementary Figure 4**).

### 3.6 Genus-level differential abundance

**Figure 8:**
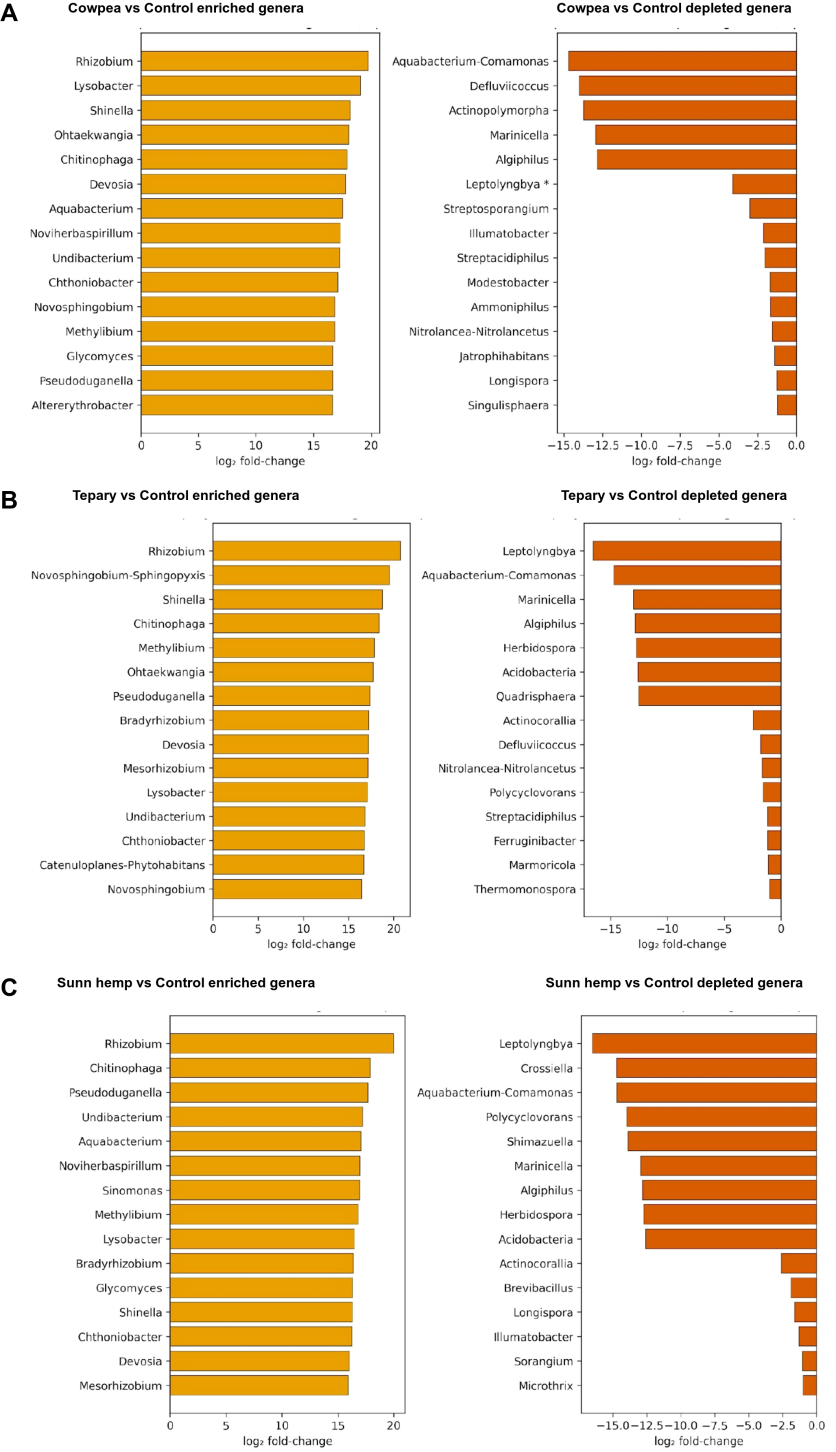
Genus-level differential abundance. Negative binomial GLMs were fit to ASV-derived counts collapsed to genus level; significance controlled by Benjamini–Hochberg FDR (q < 0.10 unless stated). Panels show contrasts of each legume vs. fallow using the same model family; effect sizes are log2 fold changes. n = 3 per treatment.

Negative binomial modelling of ASV counts identified several genera that responded consistently to cover cropping compared with fallow soil (**Figure 8**). Across pooled contrasts (all cover crops vs fallow), rhizosphere communities showed enrichment for genera associated with nitrogen cycling and rhizosphere colonization, including Azotobacter, Ensifer, and Massilia, as well as taxa linked to iron cycling and biofilm formation, such as *Leptothrix*. Additional genera involved in organic matter decomposition and nutrient turnover, including *Actinoplanes* and *Opitutus*, were also enriched under cover crop treatments. In contrast, fallow soils showed higher abundance of phototrophic or oligotrophic taxa such as *Leptolyngbya*.

Pairwise comparisons indicated similar overall patterns among the three legume species, although some crop-specific differences were observed (**Figure 8**). For instance, cowpea and tepary rhizospheres showed enrichment of *Azotobacter*, *Ensifer*, and *Massilia*, while sunn hemp further supported enrichment of taxa such as *Oxalicibacterium*. Despite these differences, the recurrence of nitrogen-associated genera (*Azotobacter*, *Ensifer*) and early rhizosphere colonizers (*Massilia*, *Leptothrix*) across treatments suggests a common shift towards microbial groups involved in nutrient cycling, residue decomposition, and rhizosphere resource utilization under legume cover cropping.

### 3.7 LEfSe biomarkers and predicted functional capacity.

**Figure 9:**
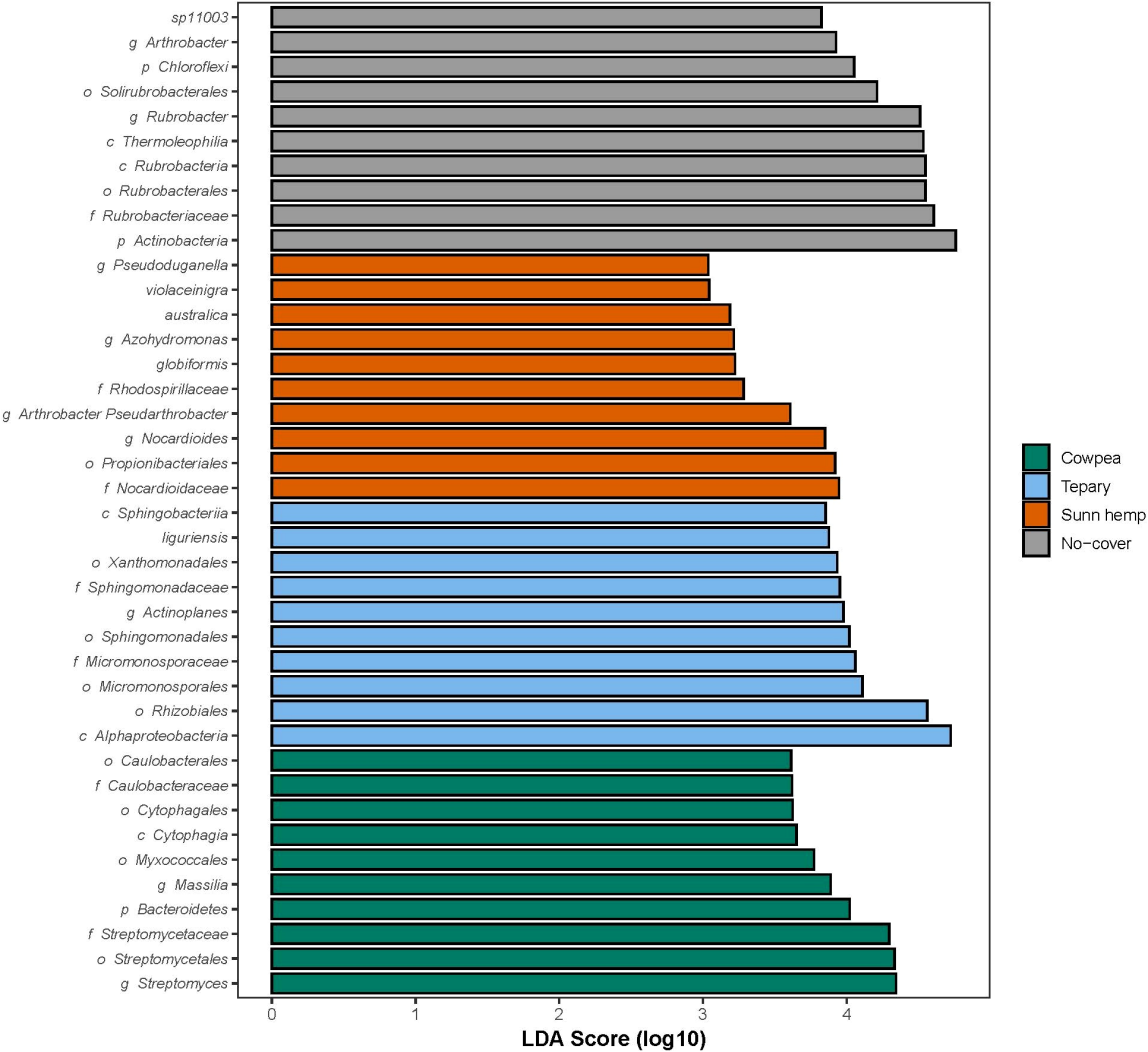
LEfSe biomarkers discriminating treatments. Kruskal–Wallis p < 0.05, pairwise Wilcoxon among subclasses, and LDA > 3. Bars indicate taxa with positive LDA scores characteristic of each group. n = 3 per treatment.

LEfSe identified discriminant taxa for each treatment (LDA > 3, p < 0.05) (**Figure 9**). Rhizobiales and Sphingomonadales characterized Tepary; cowpea by Streptomycetaceae, *Myxococcales*, and *Massilia*; sunn hemp by *Propionibacteriales* and *Micrococcales*; while fallow soils were enriched in *Rubrobacteria*, *Thermoleophilia*, and *Chloroflexi*. At finer resolution, *Rhizobium* and *Bradyrhizobium* were observed in tepary; cowpea showed signatures for Bacteroidetes, *Streptomyces*, and Oxalobacteraceae; sunn hemp exhibited *Propionibacteriales*, *Pseudoarthrobacter*, and *Methylobacteriaceae*. These biomarkers align with the community trends described above and support species-specific selection superimposed on a strong, shared rhizosphere effect of living roots.

PICRUSt2 predictions were used to infer potential functional profiles of rhizosphere microbial communities based on 16S rRNA gene sequences. Sample-weighted nearest sequenced taxon index (NSTI) values averaged 0.213 ± 0.026, with 49.4% of ASVs ≤ 0.20, indicating that reference genomes reasonably represented most sequences. All samples passed default PICRUSt2 quality checks and were retained for downstream analyses.

**Figure 10:**
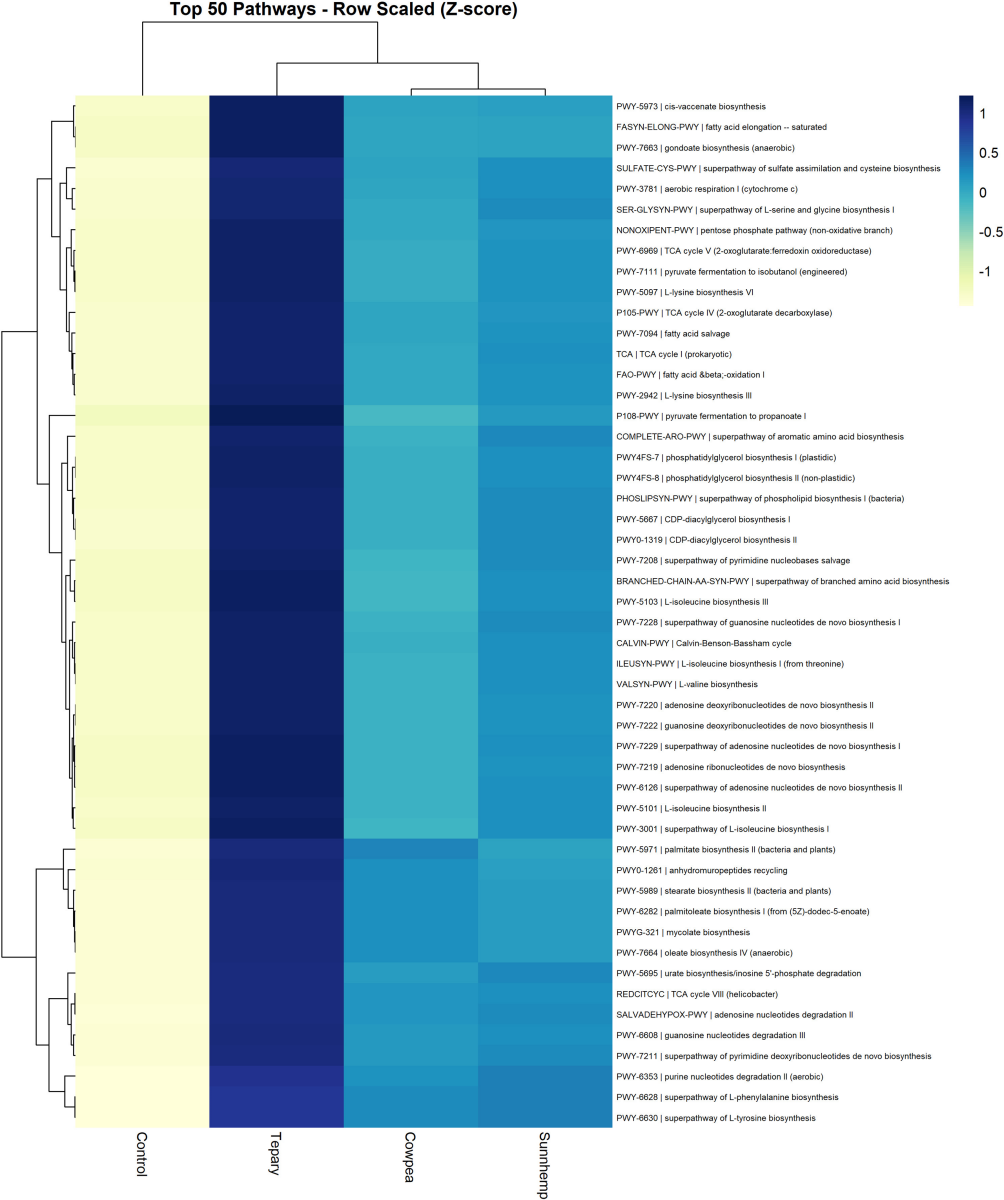
Predicted functional profiles (MetaCyc) from PICRUSt2. Heatmap shows the top 50 pathways by mean abundance across groups; values are row-scaled z-scores (blue = enriched, yellow = depleted). Columns report treatment means (n = 3 per treatment). Values reflect predicted relative capacity, not measured process rates.

MetaCyc pathway profiles showed separation between legume rhizosphere communities and fallow soils (**Figure 10**), and the top 50 predicted pathways clustered broadly by treatment group. Relative z-score patterns suggested that many biosynthetic and energy-related pathways were comparatively more represented in rhizosphere communities than in fallow soils, including pathways associated with central carbon metabolism, amino acid biosynthesis, nucleotide biosynthesis, fatty acid and lipid biosynthesis, and siderophore production (**Figure 10**; **Supplementary Figures 5**, **6**). Predicted pathways related to nitrogen metabolism, including denitrification and assimilatory nitrate reduction, also tended to show higher relative representation in cover crop rhizospheres, particularly in tepary bean samples.

However, no individual pathway remained statistically significant after false discovery rate (FDR) correction (q < 0.10) in the Kruskal–Wallis tests. Therefore, these patterns were interpreted cautiously as potential functional differences rather than confirmed evidence of altered microbial metabolic activity. Confirmation of these trends would require approaches such as shotgun metagenomics or meta-transcriptomics to directly link pathways with taxa and quantify microbial activity in the rhizosphere.

## 4 Discussion

### 4.1 Rhizosphere compositional shifts under legume cover crops.

Across the organic field setting, all three warm-season legumes produced a pronounced rhizosphere effect relative to fallow soil, with abundance-weighted phylogenetic structure capturing most of the between-group variation. This outcome aligns with the well-established view that plant roots influence microbial assembly through the release of root exudates and other rhizo-depositions that act as ecological filters in the rhizosphere. Root exudates, including sugars, amino acids, organic acids, and secondary metabolites, provide readily available carbon sources that enrich copiotrophic and root-associated taxa while reducing the relative dominance of oligotrophic groups in unplanted soils (Berendsen et al., 2012; Ling et al., 2022; Compant et al., 2024). Within this broader signal, directional differences among each legume were observed: tepary bean tended to show higher relative abundance of *Rhizobiales* (11.83%), consistent with recruitment of legume-associated nitrogen-fixing taxa; cowpea showed greater representation of Streptomyces and *Burkholderiales* linked to biocontrol and residue decomposition; and sunn hemp supported diverse *Actinobacteriota* linked to carbon turnover. These patterns suggest host-mediated recruitment processes in which plant genotype, root architecture, developmental stage, and exudate composition interact to shape rhizosphere community assembly (Pérez-Jaramillo et al., 2017; Park et al., 2023; Wagner, 2024; Ponsford et al., 2022).

### 4.2 Alpha- and beta-diversity: magnitude, drivers, and ecological interpretation

Alpha-diversity trends suggested that living roots increased richness and evenness relative to fallow. However, differences were not significant under the current replication—a result consistent with meta-analytic evidence for modest but directionally positive effects of cover crops on diversity (Kim et al., 2020). In contrast, beta-diversity contrasts were strong and consistent across metrics, with the most pronounced separation in abundance-weighted phylogenetic space. This implies that treatment effects were driven primarily by changes in the relative abundance of phylogenetically related taxa, rather than by wholesale presence–absence turnover. Such behavior is expected in carbon-rich root zones, where exudates intensify microbial activity within narrow spatial bands, creating hotspots that scale to community-level differences (Becker and Holz, 2021; Kuzyakov and Razavi, 2019). One interpretation is that, under water limitation, spatial heterogeneity in moisture can supersede root hair-mediated exudation in shaping activity (Zhang et al., 2022), providing a plausible explanation for similar alpha diversity among legumes but a stronger, abundance-weighted separation from fallow.

### 4.3 Microbial groups and consistently enriched taxa: early colonizers and N-relevant partners

Genus-level differential abundance analyses revealed a convergent set of microbial taxa across cover crops versus fallow, including *Massilia*, *Ensifer* (syn. *Sinorhizobium*), and *Azotobacter*. Independent studies increasingly identify *Massilia* as a fast-responding rhizosphere copiotroph associated with lateral root formation, flowering time, rhizosheath development, and growth promotion—root traits that enhance resource capture under stress (Wang et al., 2024; He et al., 2025). The recurrent enrichment of *Massilia*, together with diazotrophs (*Ensifer*, *Azotobacter*), supports a mechanism in which legume roots recruit early colonizers and N-relevant partners that accelerate nutrient turnover and may interface with plant developmental programs. These observations cohere with the broader concept of microbiome heritability, in which host traits and genotype govern reproducible features of rhizosphere assembly (Wagner, 2024; Ponsford et al., 2022).

### 4.4 Functional capacity: predicted pathways, ecological coherence, and cautious interpretation

PICRUSt2-based predictions indicated potential improvements in biosynthetic and energy-related pathways in legumes (central carbon metabolism, amino acid and nucleotide biosynthesis, fatty acid/lipid biosynthesis) and enhanced nitrogen transformation potential (denitrification; assimilatory nitrate reduction), with tepary bean showing the strongest signal. These directional outcomes are ecologically coherent with exudate-mediated activation of rhizosphere metabolism and recent demonstrations that cover crop exudates can redirect microbiome functional trajectories—including N cycling and phytohormone metabolisms (Seitz et al., 2024; Pang and Xu, 2024; Compant et al., 2024). Nevertheless, amplicon-based functional inference has known limitations beyond human-associated microbiomes; differentially abundant functions may diverge from shotgun benchmarks (Sun et al., 2020). Updates to the PICRUSt2 reference genome database (PICRUSt2-SC) improve representation of environmental taxa and prediction fidelity, but confirmation via shotgun metagenomics and metatranscriptomics remains essential to apportion pathways to taxa and quantify activity (Wright and Langille, 2025; Sun et al., 2020). We therefore interpret the functional results as hypothesis-generating and complementary to robust taxonomic signals.

### 4.5 Tepary bean as a climate-resilient recruiter of N-relevant taxa

A key contribution of this work is the field-based, side-by-side comparison of three warm-season legumes that are relevant to organic systems but unevenly characterized, especially tepary bean. Tepary’s tendency toward enrichment of *Rhizobiales* and the predicted increases in N pathways point to a potential capacity to stimulate rhizosphere N turnover. Recent depth-resolved analyses show that legume impacts on N-fixation genes are concentrated in the upper soil layer—precisely where exudation is maximal—reinforcing the value of rhizosphere-focused sampling (Tao et al., 2024). In the broader context of climate resilience, tepary is increasingly recognized for its superior heat and drought-resistance, and emerging studies link microclimate and soil amendments to shifts in microbial composition and functional potential, with consequences for yield and bean quality (Traub et al., 2017; Singh et al., 2024). Our results, therefore, nominate tepary as a potential climate-ready cover crop for warm-season organic systems where N supply, water limitation, and soil function must be simultaneously optimized (Tao et al., 2024; Compant et al., 2024).

### 4.6 Implications for cover crop selection and microbiome engineering

By demonstrating that summer legumes restructure the rhizosphere toward microbial groups associated with decomposition, N cycling, and potential biocontrol, our results suggest a microbial foundation for soil-health benefits observed under cover crops and consistent with meta-analytic findings of enhanced microbial abundance and activity (Kim et al., 2020). Practical implications include: (i) species-aware selection—tepary for potential N-relevant recruitment; cowpea and sunn hemp for taxa linked to carbon turnover and disease suppression—and (ii) the design of synthetic consortia or rhizosphere transplants may enrich for *Massilia*, *Ensifer*, and *Azotobacter* to accelerate early root colonization, siderophore-mediated micronutrient acquisition, and biological N inputs under heat and water stress (Wang et al., 2024; He et al., 2025; Compant et al., 2024). These strategies align with the emerging agenda to harness plant–microbiome interactions for sustainable production while acknowledging context-dependence and the need for robust field validation (Compant et al., 2024). However, we recognize the limitations that might warrant emphasis, such as (1) even if we pooled multiple plant samples, replication per treatment was modest, limiting power to detect alpha-diversity differences and some pairwise contrasts; even so, omnibus tests and effect sizes consistently supported treatment separation; and (2) functional predictions from 16S data are inferential and subject to database coverage and placement accuracy; while recent database updates improve performance, targeted multi-omics are needed for definitive functional attribution and rate measurements (Wright and Langille, 2025; Sun et al., 2020). Together, these caveats reinforce the importance of conservative interpretation and motivate the conduct of validation experiments. Because differential-abundance methods differ in sensitivity and assumptions, especially regarding compositionality, our combined LEfSe + GLM approach offers greater robustness than either method alone. Future research using ANCOM−BC or ALDEx2 could further validate the taxa identified, aligning with best-practice guidelines (Nearing et al., 2022).

Further research should focus on the mechanisms linking root exudate chemistry to microbial recruitment and nitrogen transformations in warm-season legumes. Initially, experiments resolving exudates from tepary, cowpea, and sunn hemp should evaluate whether variations in sugars, organic acids, and phenolics predict the abundance and activity of key microbial groups, such as *Rhizobiales, Massilia, and Azotobacter*. Combining targeted metabolomics with shotgun metagenomics and metatranscriptomics will help assign pathways to specific taxa and verify functions inferred from amplicons. Next, factorial trials crossing legume species with water/temperature regimes and soil N should measure process rates using ^15N tracers—like assimilation, nitrate reduction, and denitrification—along with N_2_O fluxes, enzyme activities, and plant performance. This may clarify how environmental factors influence plant–microbe interactions. Scaling analyses incorporating depth-stratified sampling, time series, and power analysis will evaluate the generality across soils and ensure the detection of effect sizes observed here. These steps will clarify when and how warm-season legumes can reliably guide rhizosphere functions to enhance nutrient cycling and resilience in organic systems.

## 5 Conclusion

Warm-season legume cover crops were associated with consistent shifts in rhizosphere bacterial communities relative to fallow soil, with communities redirecting from oligotrophic, stress-adapted assemblages toward taxa typically associated with root-responsive environments. Although all legume species showed broadly similar patterns, species-specific signatures were observed. For example, tepary bean rhizospheres showed greater relative abundance of *Rhizobiales*, which are commonly associated with nitrogen-relevant microbial groups, while cowpea favored lineages often linked to residue decomposition and potential biocontrol. Sunn hemp supported diverse *Actinobacteria-associated* taxa potentially linked to carbon cycling. Predicted functional profiles from PICRUSt2 broadly reflected these compositional trends. Several biosynthetic and nutrient-related pathways, including central carbon metabolism, amino acid and nucleotide biosynthesis, fatty acid and lipid metabolism, siderophore production, and nitrogen-related pathways, showed higher relative representation in legume rhizospheres compared with fallow soils. However, as these predictions are based on phylogenetic inference and no pathways remained significant after false discovery rate correction, these patterns were interpreted cautiously as potential functional tendencies rather than direct evidence of altered microbial activity.

Together, these results suggest that summer legume cover crops may influence rhizosphere microbial community composition and predicted functional potential in organic production systems. Among the species evaluated, tepary bean showed microbial community patterns comparable to those of commonly used summer legumes. It may represent a promising option for warm-season cover cropping in southern Texas. These findings highlight potential opportunities to explore how species selection may shape rhizosphere microbial communities and associated ecological functions in organic systems. The recurrent appearance of genera such as *Massilia*, *Ensifer*, and *Azotobacter*, which are commonly associated with rhizosphere colonization and nutrient cycling, suggests possible microbial groups of interest for future investigation. However, further studies incorporating larger sample sizes and direct functional approaches, such as shotgun metagenomics and metatranscriptomics, will be required to validate microbial functions and clarify the ecological mechanisms underlying these patterns.

## Data Availability

The datasets presented in this study can be found in online repositories. The names of the repository/repositories and accession number(s) can be found below: https://www.ncbi.nlm.nih.gov/sra/?term=PRJNA1337659.

## Supplementary material

Supplementary Figure 1 Rarefaction curves (Chao1) from the rarefied ASV table; n = 3 per treatment. Curves plateau at ≥12,000 reads, indicating adequate sampling depth.

Supplementary Figure 2 PCoA of Bray–Curtis dissimilarities from the rarefied ASV table; ellipses = 95% confidence intervals; n = 3 per treatment. **(A)** All treatments: PC1 = 34.95%, PC2 = 13.19%. PERMANOVA (999 permutations): F = 4.28, R² = 0.30, p = 0.006. **(B)** Cover crops vs. fallow (same test parameters).

Supplementary Figure 3 PCoA of unweighted UniFrac distances from the rarefied ASV table; ellipses = 95% confidence intervals; n = 3 per treatment. **(A)** All treatments: PC1 = 26.76%, PC2 = 20.50%. Reported PERMANOVA parameters as in main text.

Supplementary Figure 4 Relative abundance bar plots at additional taxonomic ranks. **(A)** Class, **(B)** Family, **(C)** Genus, **(D)** Species. Top 25 taxa shown per rank; remaining aggregated as “Other.” n = 3 per treatment.

Supplementary Figure 5 MetaCyc superclass heatmap (PICRUSt2). Columns are treatment means (n = 3 per treatment). Row-scaled z-scores (green = enriched, brown = depleted). Values represent predicted relative capacity.

Supplementary Figure 6 Predicted pathway categories from PICRUSt2: **(A)** Carbohydrate metabolism, **(B)** Fatty acid/lipid biosynthesis, **(C)** Siderophore biosynthesis, **(D)** Nitrogen metabolism, **(E)** Amino acid biosynthesis, **(F)** Nucleotide biosynthesis. Bars show treatment means ± SD (n = 3 per treatment). Pathways reflect predicted relative capacity.

Supplementary Table 1 Biomass of cover crop species and soil nitrate values before sowing and after decomposition of cover corps.

Supplementary Table 2 Read processing summary for 16S rRNA amplicon data across cowpea, tepary bean, sunn hemp, and fallow soil samples including raw reads, trimmed reads, DADA2-inferred sequences, chimeric sequences, chimera-free reads, and final ASV counts after size filtration.

Supplementary Table 3 Differences in alpha diversity (Chao1) richness measured using the Kruskal-Walli’s test. Benjamini–Hochberg–adjusted Dunn’s *post hoc* test was conducted to compare cover crops with a no-cover control and within each cover crop. Differences were minimal and not statistically significant. Effect sizes were measured using Cliff’s delta (δ range −1.00 to −0.56) and Hedges’ g (g range −1.30 to −1.25).

Supplementary Table 4 Homogeneity of multivariate dispersion (betadisper) results for microbial community structure based on weighted UniFrac distances. ANOVA results, permutation tests (999 permutations), and Tukey HSD pairwise comparisons of distances to group centroids are shown for cowpea, tepary bean, sunn hemp, and fallow soil treatments.
