# Supplementary figures and images for: Functional and taxonomic shifts in rhizosphere microbiomes of summer legume cover crops
Source: Front Plant Sci. 2026 May 1;17:1790810. doi: 10.3389/fpls.2026.1790810 (PMC13178294; doi:10.3389/fpls.2026.1790810)

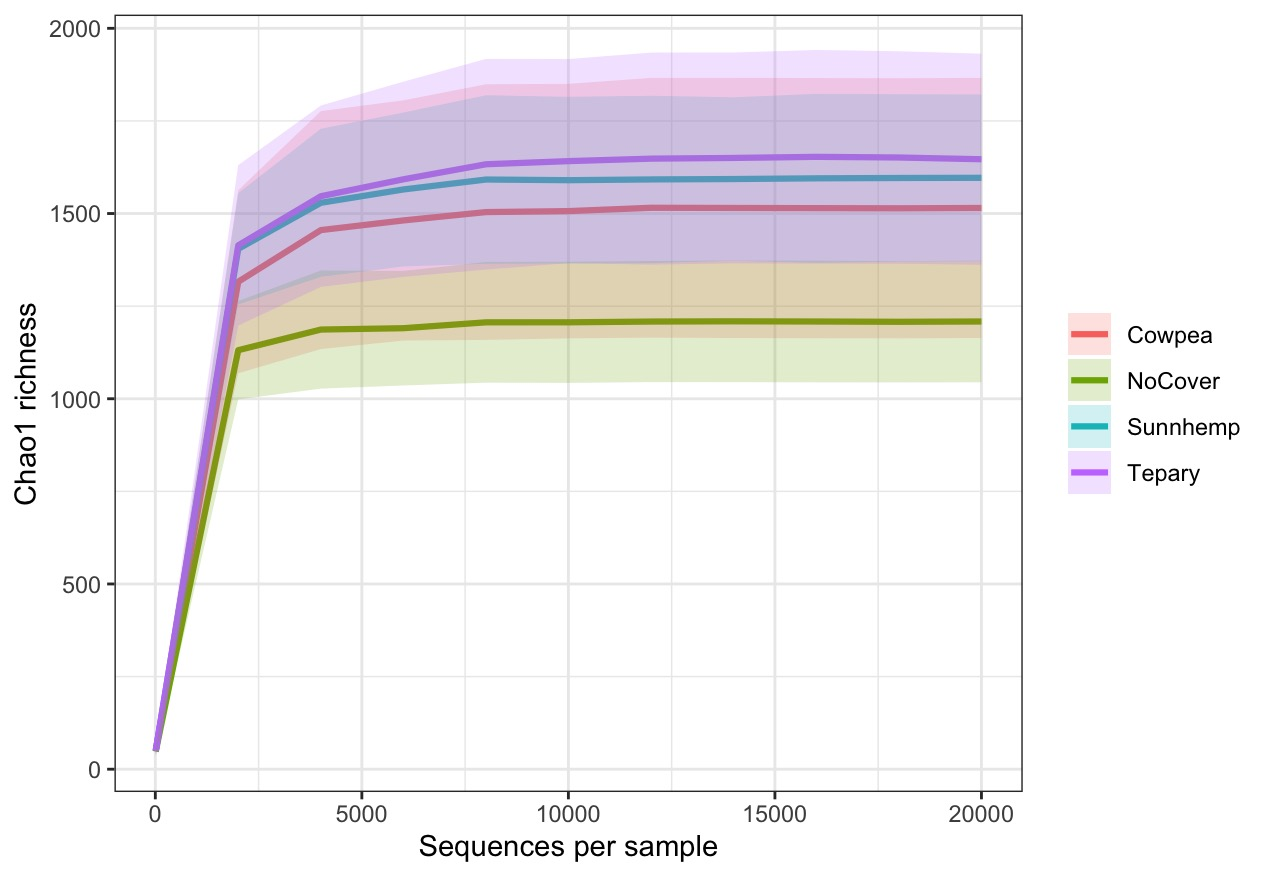

Supplement: Supplementary Figure 1 — Rarefaction curves (Chao1) from the rarefied ASV table; n = 3 per treatment. Curves plateau at ≥12,000 reads, indicating adequate sampling depth. [file Image1.tiff]

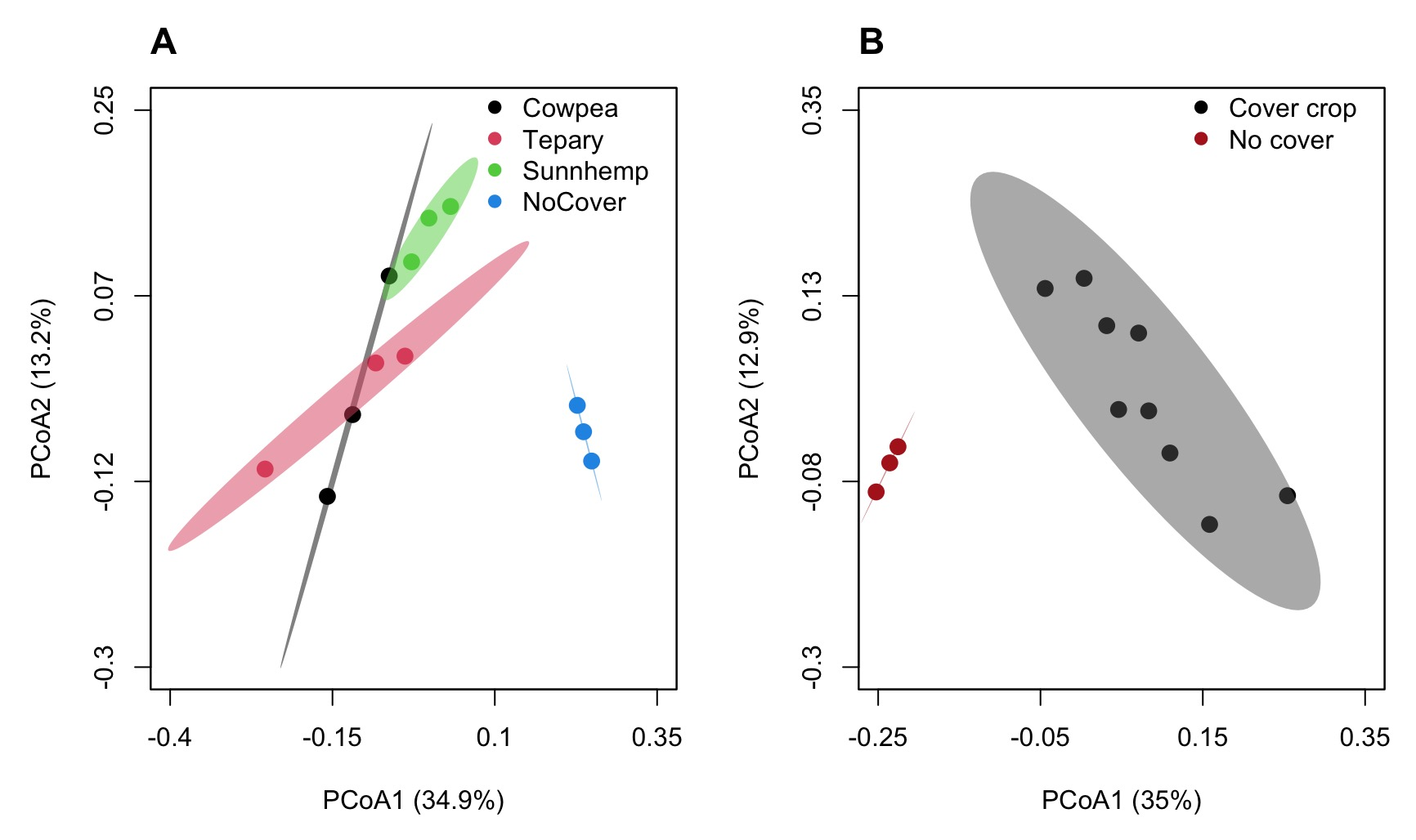

Supplement: Supplementary Figure 2 — PCoA of Bray–Curtis dissimilarities from the rarefied ASV table; ellipses = 95% confidence intervals; n = 3 per treatment. (A) All treatments: PC1 = 34.95%, PC2 = 13.19%. PERMANOVA (999 permutations): F = 4.28, R² = 0.30, p = 0.006. (B) Cover crops vs. fallow (same test parameters). [file Image2.tiff]

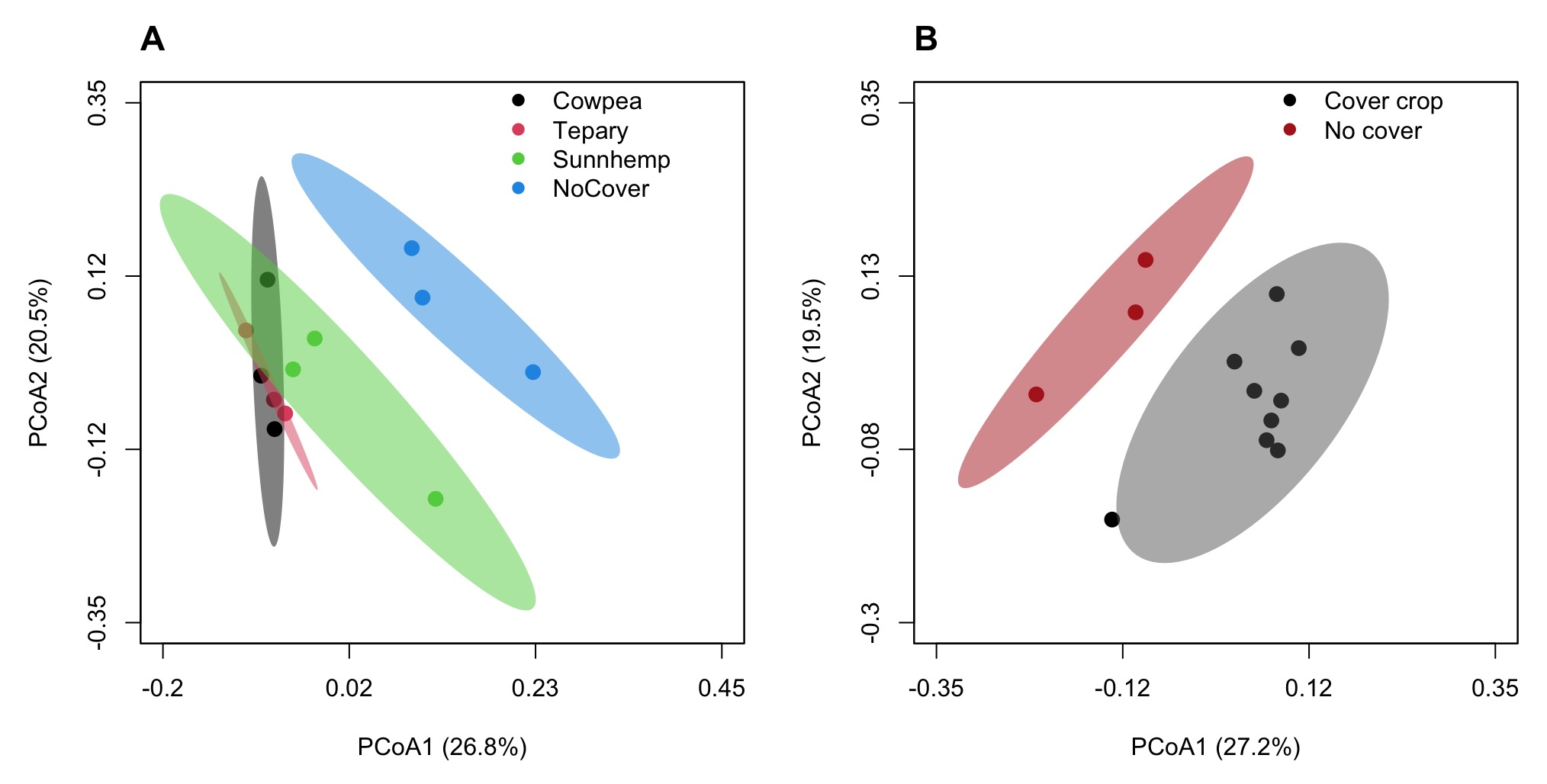

Supplement: Supplementary Figure 3 — PCoA of unweighted UniFrac distances from the rarefied ASV table; ellipses = 95% confidence intervals; n = 3 per treatment. (A) All treatments: PC1 = 26.76%, PC2 = 20.50%. Reported PERMANOVA parameters as in main text. [file Image3.tiff]

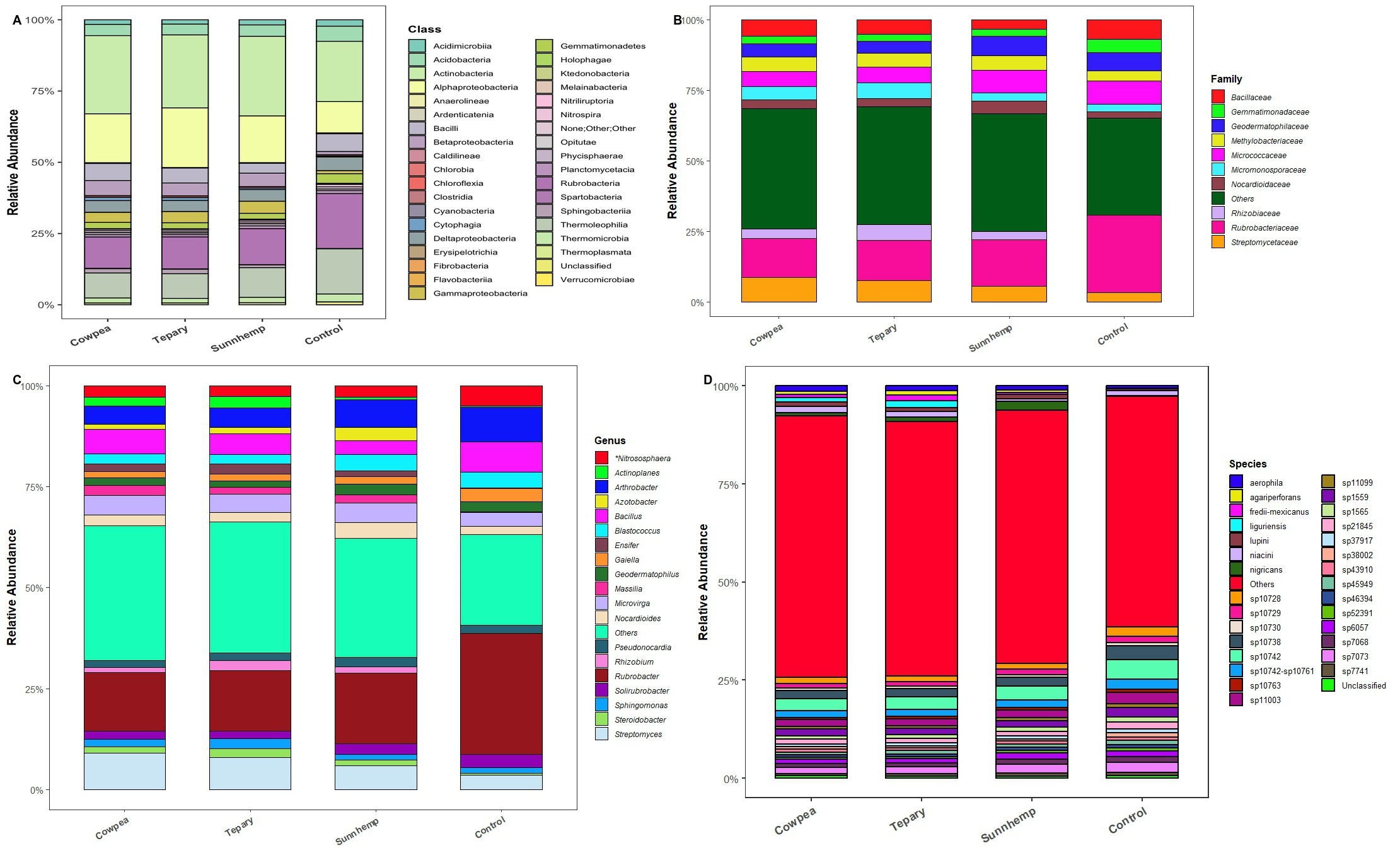

Supplement: Supplementary Figure 4 — Relative abundance bar plots at additional taxonomic ranks. (A) Class, (B) Family, (C) Genus, (D) Species. Top 25 taxa shown per rank; remaining aggregated as “Other.” n = 3 per treatment. [file Image4.tiff]

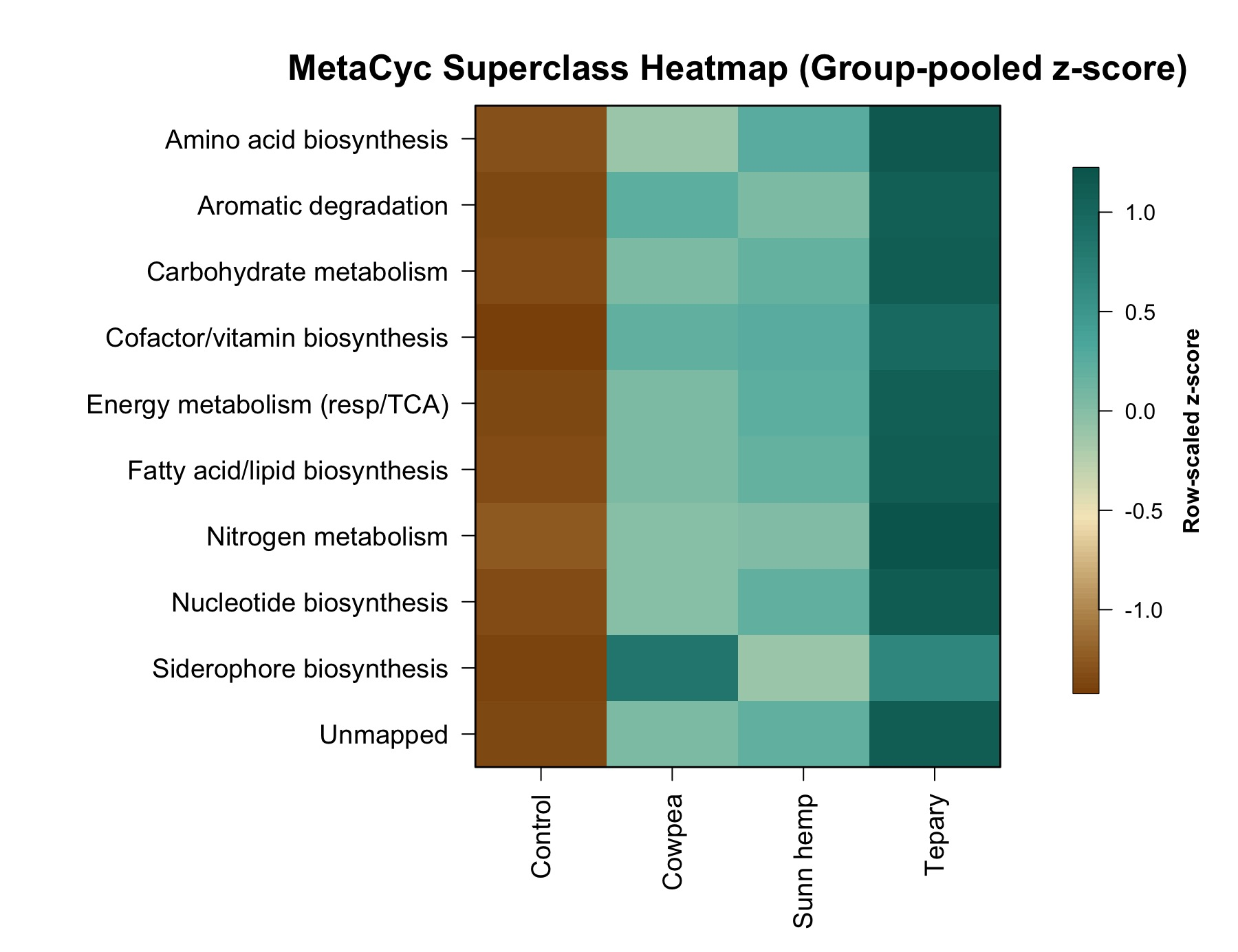

Supplement: Supplementary Figure 5 — MetaCyc superclass heatmap (PICRUSt2). Columns are treatment means (n = 3 per treatment). Row-scaled z-scores (green = enriched, brown = depleted). Values represent predicted relative capacity. [file Image5.tiff]

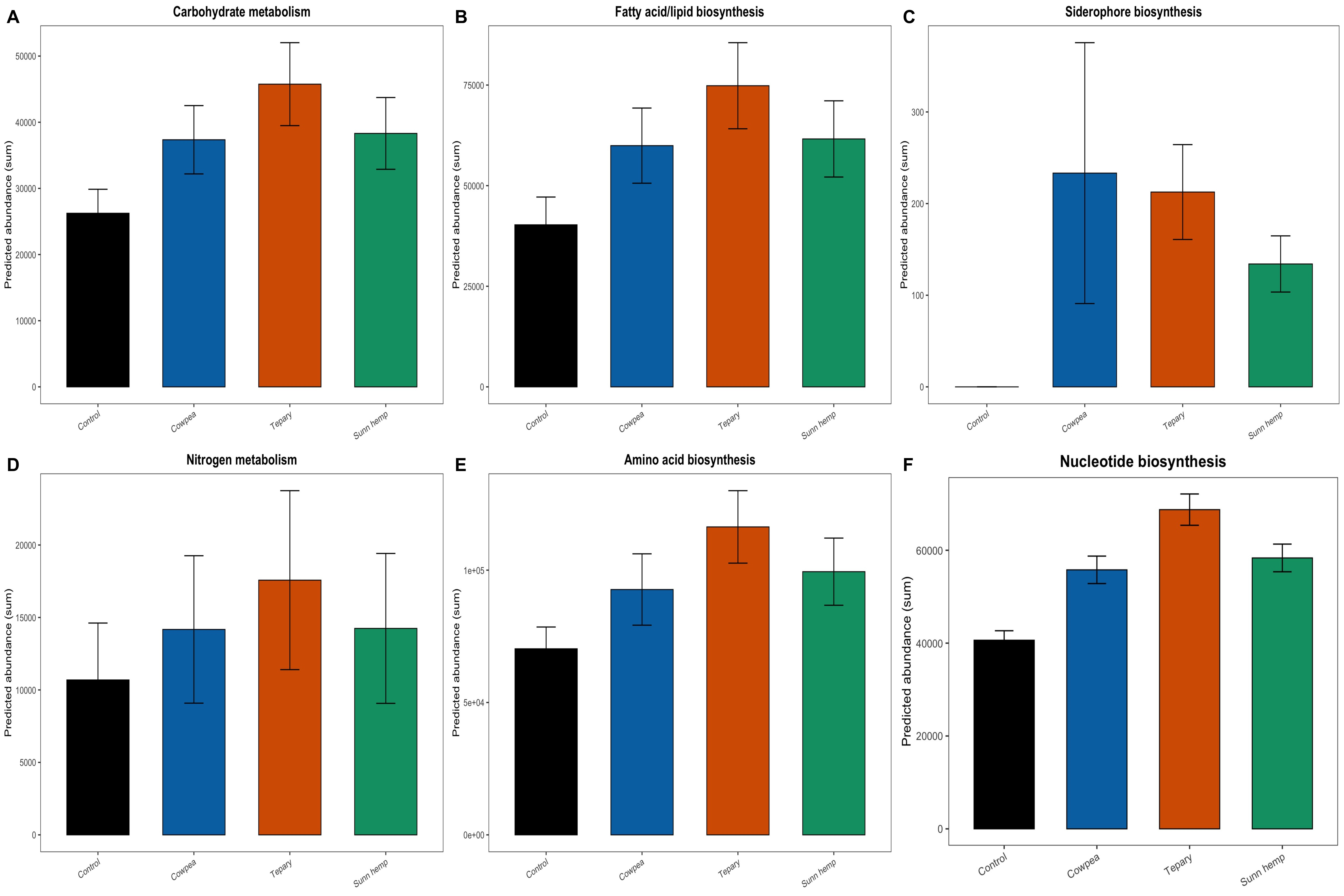

Supplement: Supplementary Figure 6 — Predicted pathway categories from PICRUSt2: (A) Carbohydrate metabolism, (B) Fatty acid/lipid biosynthesis, (C) Siderophore biosynthesis, (D) Nitrogen metabolism, (E) Amino acid biosynthesis, (F) Nucleotide biosynthesis. Bars show treatment means ± SD (n = 3 per treatment). Pathways reflect predicted relative capacity. [file Image6.tiff]
